# Coding of level of ambiguity within neural systems mediating choice

**DOI:** 10.3389/fnins.2013.00229

**Published:** 2013-12-06

**Authors:** Dan Lopez-Paniagua, Carol A. Seger

**Affiliations:** Department of Psychology, Colorado State UniversityFort Collins, CO, USA

**Keywords:** ambiguity, risk, uncertainty, probability distribution, probabilistic outcome prediction, fMRI, BOLD

## Abstract

Data from previous neuroimaging studies exploring neural activity associated with uncertainty suggest varying levels of activation associated with changing degrees of uncertainty in neural regions that mediate choice behavior. The present study used a novel task that parametrically controlled the amount of information hidden from the subject; levels of uncertainty ranged from full ambiguity (no information about probability of winning) through multiple levels of partial ambiguity, to a condition of risk only (zero ambiguity with full knowledge of the probability of winning). A parametric analysis compared a linear model in which weighting increased as a function of level of ambiguity, and an inverted-U quadratic models in which partial ambiguity conditions were weighted most heavily. Overall we found that risk and all levels of ambiguity recruited a common “fronto—parietal—striatal” network including regions within the dorsolateral prefrontal cortex, intraparietal sulcus, and dorsal striatum. Activation was greatest across these regions and additional anterior and superior prefrontal regions for the quadratic function which most heavily weighs trials with partial ambiguity. These results suggest that the neural regions involved in decision processes do not merely track the absolute degree ambiguity or type of uncertainty (risk vs. ambiguity). Instead, recruitment of prefrontal regions may result from greater degree of difficulty in conditions of partial ambiguity: when information regarding reward probabilities important for decision making is hidden or not easily obtained the subject must engage in a search for tractable information. Additionally, this study identified regions of activity related to the valuation of potential gains associated with stimuli or options (including the orbitofrontal and medial prefrontal cortices and dorsal striatum) and related to winning (including orbitofrontal cortex and ventral striatum).

## Introduction

Making decisions is an integral part of everyday life, yet, the factors affecting our decisions are not fully known. One important parameter of decision making is the degree of uncertainty present in any given choice. Most theories of decision making postulate that decision making involves two important functions: valuation, in which the agent calculates the likely benefits associated with each option, and choice, in which the expected gains are modulated by other factors to determine the final decision (Montague et al., 2006; Vlaev et al., 2011). Choices can vary greatly in the level of information about the distribution of potential outcomes available to the decision-making agent. In some uncertain choices, such as gambling on the outcome of a roulette game, probability can be easily determined from relative frequencies of likely, or past outcomes. In other uncertain choices probabilities are based on incomplete or missing information, such as in deciding whether to bring an umbrella in case of rain. In economics, these two types of uncertainty have been termed “risk” and “ambiguity” respectively (Knight, 1921; Ellsberg, 1961), and have been dissociated behaviorally across numerous studies (Sanfey and Chang, 2008).

For this study, we used the term “uncertainty” as an umbrella term to refer to situations where the outcome or probability is not certain, which includes both risk and ambiguity. For example, risk can be seen as knowable information readily available to subjects regarding the probability of a desired outcome, yet the outcome itself is certain (Knight, 1921). Alternately, ambiguity can be defined as unknown information (that is either hidden or not readily available to subjects) regarding the probability of a favorable outcome (Knight, 1921; Camerer, 1995; Bach et al., 2011).

Within the past decade, several studies made significant breakthroughs in understanding the neural mechanisms underlying uncertainty processing in decision making tasks involving economic (Hsu et al., 2005; Rustichini et al., 2005; Brand et al., 2006; Huettel et al., 2006) and contextual (Hsu et al., 2005) uncertainty. These studies found more activity for ambiguity than for risk in the dorsolateral prefrontal cortex (DLPFC), parietal cortex, and dorsal striatum. These regions are typically recruited across a variety of decision making tasks and we will refer to them as the “fronto–parietal–striatal system.” In addition these studies found activity in the insula thought to be related to the aversive nature of ambiguity.

More recently, there has been a movement away from limited, categorical definitions of uncertainty (particularly ambiguity), in which uncertainty is treated as an all-or-nothing variable. Although that approach proved powerful in dissociating the neural substrates of ambiguity from risk, it precludes examination of decision making in response to different degrees of ambiguity. Individuals rarely experience real-life situations where information regarding the probability of reward is an absolute unknown; therefore, the question of how neural signals are modulated by partial uncertainty is an important one.

Behavioral studies have previously explored decision making under conditions of partial ambiguity in attempts to better understand ambiguity aversion (Larson, 1980; Keren and Gerritsen, 1999). These studies defined ambiguity using the same definition that we use: as missing, yet potentially knowable, information regarding the probability of a certain outcome (Camerer, 1995). Overall they found that “perceived informativeness,” or the person's beliefs about the availability of recoverable hidden information, is a major component in determining the level of ambiguity in any given decision, regardless of the actual information presented or hidden.

Recently, several neuroimaging studies of note have examined neural responses in conditions of partial ambiguity. Levy et al. (2010) found that subjective value signals in the prefrontal cortex and striatum were influenced by both risk and ambiguity, and activity in the orbitofrontal cortex (OFC) was influenced by partial ambiguity. Bach et al. (2009) examined neural responses to different levels of ambiguity within a choice-free conditioning task and found increased activation in the DLPFC for partial ambiguity compared to no ambiguity (risk) and full ambiguity (ignorance) conditions. They argued this frontal region was involved in extracting relevant information from the learning context, which is most necessary and helpful during intermediate levels of ambiguity. In a follow-up study using an instrumental avoidance learning task, Bach and colleagues (2011) continued their examination of the effects of risk and ambiguity on neural signals by modeling ambiguity as a continuous variable, via measures of entropy of reward probabilities, in which conditions of partial ambiguity were associated with higher entropy. Bach et al. (2011) again found activation along a frontal, parietal, and striatal regions associated with partial ambiguity. More importantly, Bach and colleagues (2011) found that the effects of partial ambiguity were not driven by second-order uncertainty (ambiguity) alone.

The current study introduced a cognitive decision making task that included multiple levels of ambiguity. Like Bach et al. (2011), we focused primarily on ambiguity-related signals. In our task, players chose to play either a lottery that varied in the amount of uncertainty displayed or a constant (or reference) lottery and experienced monetary gains and losses instead of the instrumental avoidance with pain reinforcement task used by Bach and colleagues (2011). Unlike Bach and colleagues (2011), who manipulated ambiguity as a continuous function, we relied on a graded measure of ambiguity. We examined 6 levels of ambiguity, ranging from zero ambiguity (risk), through 4 levels of partial ambiguity, to full ambiguity (ignorance). By controlling the amount of ambiguity presented to subjects, we were able to similarly explore if incrementally increasing levels of ambiguity elicit a similarly graded response in regions of the brain previously identified as being sensitive to uncertainty (Bach et al., 2009, 2011), such as the DLPFC, parietal cortex and striatum.

We examined two hypothetical relationships between neural activity and ambiguity. The first hypothesis was that neural activity would increase monotonically as ambiguity increased, and is consistent with studies finding overall patterns of greater activity during ambiguity than during risk (see Krain et al., 2006). A second hypothesis was that there would be an inverted-U relationship between degree of ambiguity and neural activity; this hypothesis was based on the findings of Bach and colleagues (2009, 2011) that intermediate levels of uncertainty elicited greater activation in fronto–parietal–striatal regions than did conditions of no uncertainty and full uncertainty in a choice free conditioning task. To examine both hypotheses, we modeled ambiguity as both linearly increasing and inverted-U quadratic functions.

We predicted that the inverted-U function would best account for activity in fronto–parietal–striatal regions. Our rationale was based on the theory put forward by Bach et al. (2009) that intermediate levels of ambiguity lead to an increased search for hidden but searchable information. Decisions on the basis of complete information (as in risk), or very little information (as in full ambiguity), can be made with minimal processing without placing significant demands on cognitive control functions within the DLPFC, and can be mediated by relatively posterior motor regions of the frontal lobe. However, as decisions become “harder” as in situations with intermediate ambiguity, anterior regions of the DLPFC should be recruited to provide supplemental processing as the demands to extract contextual information increase.

Besides examining the direct effects of partial ambiguity, we explored how the brain tracks expected reward in various states of uncertainty. We hypothesized that uncertainty would modulate activity in neural regions associated with reward, and reward-related activity would decrease as uncertainty increased. We expected to find this pattern within OFC (Padoa-Schioppa and Assad, 2006; Plassmann et al., 2007), dorsal striatum (Samejima et al., 2005; Lau and Glimcher, 2008; Hsu et al., 2009), superior parietal cortex (Churchland et al., 2008; Wang, 2008), and dorsomedial prefrontal cortex (Knutson et al., 2003; Xue et al., 2009). Our design also allowed us to examine regions of activity associated with winning money. Broadly, we expected there would be greater activity within regions associated with reward representation for wins than losses. We were particularly interested in examining win related activity within the ventral striatum and how that activity may be better reflected by a non-linear increase in choice-related uncertainty. We predicted that overall the ventral striatum would be more active for winning money in comparison with losing money, but only for trials in which value could be clearly tracked, like situations involving low or high levels of uncertainty. Given this region's sensitivity to deviations from expectation, formally known as reward prediction error (see Schultz, 2002), we also predicted increased activity in the ventral striatum for unexpected wins (e.g., money won via the variable lottery) compared to expected wins (money won via the safe lottery).

## Methods

### Participants

Scanned participants included 14 healthy, right-handed young adults (age range = 22–36, mean age = 26.8 years; 5 males, 9 females) recruited from the Colorado State University (Fort Collins, CO) and University of Colorado, Denver (Aurora, CO) communities. We set 22 years as the minimum age rather than the more typical 18 based on studies showing that frontal lobe development is not complete until the early 20 s (see Hedden and Gabrieli, 2004). All participants were fluent speakers of English and were screened for a history of neurological and psychiatric disorders, and contraindications to MRI (i.e., no metallic implants, no claustrophobia, head size compatible with RF coil). Participants were pre-screened for uncertainty preference by using a shortened version of our task in a training session outside the scanner. We excluded potential participants who fell at either extreme (Total of 6 not scanned): both those with strong uncertainty preference (defined as choosing to play the variable lottery 75% or more of the time) and those with strong uncertainty aversion (defined as choosing to play the uncertain lottery less than 20% of the time). The Colorado State University institutional review board approved the experimental protocol, and written informed consent was obtained from all subjects.

### Task

**Figure 1:**
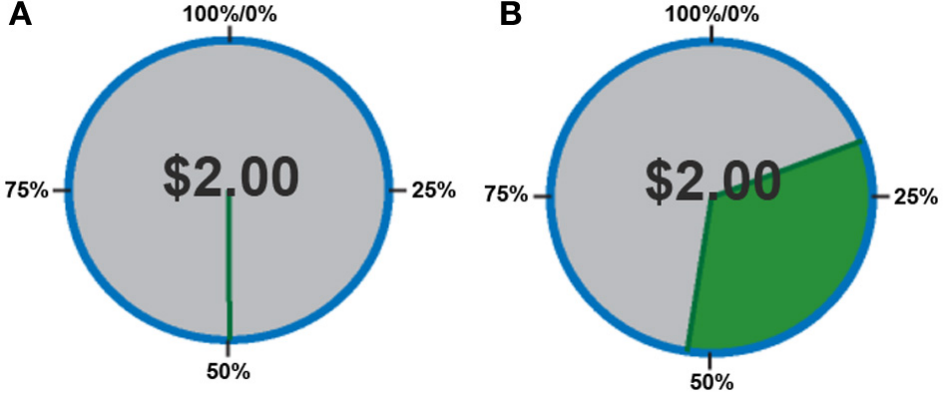
**Examples of the stimuli presented to subjects. (A)** Risky lottery (a0) showing a dial pointing to a specific probability of obtaining the sum of money in the center of the circle, and should be interpreted as 50% chance of winning $2.00. **(B)** Partially ambiguous lottery (a33). Subjects are told that the dial is hidden within the green field, suggesting a range of outcome probabilities.

In this experiment, participants performed a two-alternative, forced-choice task. The task required subjects to choose to play one of two mixed lotteries: one variable lottery that was always represented visually, and a constant (or reference) lottery that subjects were informed about but which was not represented visually on the display. The construction of the stimuli was crucial so that the variable lottery varied in the amount of information, or ambiguity, it represented. First, each lottery circle (see Figure 1A) was associated with an outcome (in this case, the amount of money that could be either won or lost) presented in the center of the circle. The probability of winning the specified amount of money was represented on the outer edge of the circle, which started at 0% in the 12 o'clock position and increased to 100% in a clockwise direction. A “dial” was then used to indicate a specific probability of winning the specified amount of money; however, this dial was hidden from view by an occluder on partial and full ambiguity trials. The size of the occlusion hiding the dial varied in size to occlude 15, 33, 66, 80, or 100% of the lottery circle (Figure 1B). The center of the occlusion was placed within ±10° of the actual winning probability. Our pilot data showed no differences between having the center of the occlusion relatively “fixed” around the winning probability and dynamically changing the position of the occlusion relative to the winning probability on every trial.

Subjects were told that although they could not see the dial, it was hidden somewhere inside the occlusion, which thereby represented a range of probabilities. By occluding the actual probability of winning, the information needed to calculate expected value was incomplete, introducing ambiguity per the classical economic definition. This manipulation allowed us to have trials in which there was no ambiguity (risk) as well as trials in which there was full ambiguity within the same task, as in Hsu et al. (2005) and Huettel et al. (2006). More importantly, this allowed us to more carefully manipulate ambiguity and examine behavioral and neural responses to parametrically increasing levels of uncertainty. One thing to note is that unlike Bach et al. (2009), in which subjects did not know how the odds in that task were constructed, due to their use of Pavlovian conditioning, subjects in our task never experienced a condition of *complete* ambiguity, which was referred to as ignorance by Bach et al. (2009). That is, subjects were able to recover *some* information regarding missing probabilities due the use of two-stage bets in our task which allowed subjects to learn about the task and how the lottery was constructed over time, rather than the complete ambiguity that would result if they were presented with novel stimuli.

Finally, the amount of money and probability of winning or losing on the variable lottery was combined so that it varied in terms of expected monetary gain in relation to the constant lottery, which was 100% chance of winning $1.00. For this study, we applied the basic idea behind expected value theory that states value can be assigned to decisions by multiplying the outcome probability with the amount of the potential pay out. We used this simplified calculation approach to calculate expected gain for each trial as the probability of winning multiplied by the potential pay out. This calculation provided a rough measure of how “good” or “bad” a decision was in relation to the potential monetary gain of the constant lottery. On certain trials, the variable lottery was constructed in a manner where the expected gain of the variable lottery was greater than the certain lottery, making these trials ambiguity advantageous (A trials). These included: 33% chance of winning $5 and 50% chance of winning $3. On specific trials, the certain lottery had a higher expected gain than the variable lottery making these trials ambiguity disadvantageous (D trials). These included: 20% chance of winning $3 and 33% chance of winning $2. Finally, some trials were set up in a manner in which the expected gain of the variable lottery matched the expected gain of the constant lottery (N trials). These included: 20% chance of winning $5, 33% chance of winning $3, or 50% chance of winning $2. Including trials in which expected gains were equal was a crucial part of this study, since they provide a quick, simple and objective measure of uncertainty sensitivity without explicitly modeling behavior. Equal numbers of each type of trial were included: 1/3 trials A, 1/3 trials D and 1/3 trials N. It is important to note that on trials in which subjects chose to play the variable lottery but did not win, they lost the amount of money indicated on this screen. We found in our pilot work developing this task that inclusion of loss was essential for obtaining a good distribution of choices within subjects. However, in our instructions to the subjects, we asked them to adopt the strategy of trying to win as much as possible. Our behavioral results are consistent with subjects having adopted a gain maximization strategy, rather than a loss avoidance strategy. If subjects had adopted a loss avoidance strategy we would have expected that they would have always chosen the safe reference lottery, even on disadvantageous trials; in actuality, no subject adopted this strategy.

Subjects were compensated at a base rate of $25 USD/hour. Subjects were also given the opportunity to add to their total pay based on performance. Before the scanning session, subjects were asked to choose 12 numbers between 1 and 180 (the number of trials in the study, unbeknownst to the subject) and told that certain trials would be chosen at random to be played for real money. After the scan, subjects were informed that these numbers corresponded to a specific trial number, and would receive the cumulative sum of their winnings from these trials as additional pay; subjects had the opportunity to win up to $60 if the trials chosen were all $5 winning lotteries, or $0 if none of the trials chosen resulted in wins. However, all subjects received at least the minimum $25 base pay. This payment mechanism ensured that subjects treated every trial as if they would be paid according to the outcome of that trial (Smith and Walker, 1993).

#### Design

For this task, subjects performed 180 trials, divided evenly into 3 scans (60 trials per scan). On each trial, the variable lottery was presented for 5 s, during which participants were required to make a response whether they chose to play the displayed lottery, or if they wanted to play the certain (not shown) lottery. Following the response, there was a short, 1.5 s window before participants were given feedback as to the outcome of their choice (either winning or losing $2, $3, or $5 dollars) for 1.5 s. After feedback, a jittered inter-trial interval, ranging between 2 and 10 s randomly sampled from a geometric distribution, was presented. This experiment used a rapid event-related fMRI design. Trials were arranged pseudo-randomly to control for any sequential effects, and “null” jittered ITI provided a measure of baseline activation (Donaldson, 2004; Bandettini, 2007). In order to perform effective connectivity analyses via Granger Causality Mapping (which were subsequently dropped from this study), all timing elements in this study summed up so that total trial length was limited to multiples of the TR, (i.e., 2, 4 s, etc), so as to keep trial onset synchronized with TR onset (Roebroeck et al., 2005). In total, the task required ~40 min of scanning time.

Visual stimuli were presented to participants using magnet-compatible goggles (Resonance Technology Inc., Los Angeles, CA). A computer running E-Prime experiment software (Psychology Software Tools Inc., Pittsburg, PA) was used to control stimulus presentation and interface with a magnet compatible response box. Head movement was minimized using a custom-fitted head holder, consisting of polyurethane foam beads inflated to tightly mold around the head and neck.

#### fMRI image acquisition

Images were obtained on a research-dedicated 3.0T whole-body MRI scanner (GE Healthcare, Milwaukee, WI) located on the campus of the University of Colorado Health.

Sciences Center, Aurora, CO. The scanner was equipped with an 8-channel, high-resolution phased array head coil using GE's Array Spatial Sensitivity Encoding Technique (ASSET) software. A trial scan of whole-brain EPI was acquired before the functional scans. Functional images were reconstructed from 31 + 5 axial oblique slices obtained using a T2^*^-weighted, volume-selective z-shim pulse sequence (*TR* = 2000 ms; *TE* = 26 ms; FA, 77°; FOV, 220-mm; 64 × 64 matrix; 4.0-mm slices; no inter-slice gap) adapted from the EPI-Gradient-Echo sequence. The z-shim pulse sequence was developed to address signal loss in neural regions adjacent to air cavities, such as the OFC. This protocol acquires additional volumes with a compensation gradient that are then combined with the original volume data to compensate for regions of signal dropout. Recently, Du and colleagues developed a sequence that minimized signal dropout in the OFC, in which the z-shim compensation is applied only to volumes that show significant signal loss, thereby substantially decreasing scanning time (Du et al., 2007). Echo-planar images from the initial trial scan were used to determine the number and location of the z-shim slices in which the OFC showed intermediate or severe SFG signal loss. Overall, five continuous slice locations were typically sufficient to cover the regions affected by the susceptibility artifacts. Anatomical images were then collected using a T1-weighted SPGR sequence (minimal TR; *TE* = 3.95 ms; *TI* = 950 ms; FA, 10°; FOV, 220-mm; 256 × 256 coronal matrix; 166 1.2-mm slices). This set of structural images was used to verify proper slice selection and to determine the sites of functional activation. Finally, functional data from the inferior cerebellum was not collected because it was necessary to adjust slice acquisition angle and the field of view (FOV) to obtain the best possible signal-to-noise ratio in the frontal lobe.

#### fMRI image analysis

Before preprocessing, functional images with and without z-shim compensation were combined using MatLab (The Mathworks, Inc. Houston, TX) using a specially written z-shim toolbox. The intensity in the composite images was multiplied by a factor of 1.33 to reduce signal discontinuity between image sets (Du et al., 2007). Image analysis was performed using BrainVoyager QX V1.10 and V2.4 (Brain Innovation, Maastricht, The Netherlands). Functional data was first subjected to preprocessing, consisting of (1) three dimensional motion correction using trilinear interpolation, (2) slice scan time correction using cubic spline interpolation, (3) temporal data filtering with a high-pass filter of 3 cycles in the time course and (4) linear trend removal. Each subject's high-resolution anatomical image was then normalized to the (Talairach and Tournoux, 1998) brain template. The normalization process consisted of two steps: an initial rigid body translation into the AC-PC plane, followed by an elastic deformation into the standard space performed on 12 individual sub-volumes. The resulting set of transformations was applied to the subject's functional image volumes to form volume time course representations to be used in subsequent statistical analyses. Finally, the volume time course representations were spatially smoothed using a Gaussian kernel, full-width at half maximum (FWHM) of 4.0 mm.

In order to identify brain regions that showed significant signal changes in response to a task demands, imaging data was analyzed using two main statistical methods. First, a whole brain analysis was performed using the general linear model (GLM) provided by BrainVoyager QX across the separate conditions. Regressors were formed by modeling the trials associated with each condition as epochs using a box-car function that were then convolved with the standard hemodynamic response function implemented in BrainVoyager QX. For analysis, experimental trials were broken into conditions on the basis of ambiguity level of the variable lottery, yielding 6 conditions: 0% ambiguity (*a0*), 15% ambiguity (*a15*), 33% ambiguity (*a33*), 66% ambiguity (*a66*), 80% ambiguity (*a80*) and 100% ambiguity (*a100*). Each ambiguity condition was presented for 30 trials. Trials were also separated into conditions based on the expected gain of the variable lottery in comparison to the constant lottery, yielding three conditions: ambiguity advantageous (*Adv*), ambiguity disadvantageous (*Disadv*) and neutral trials. We further separated our data based on whether or not subjects chose to play the variable lottery (*Uncert*) or play the constant lottery (*Cert*) yielding 6 possible conditions: *Adv-Uncert, Adv-Cert, Neutral-Uncert, Neutral-Cert, Disadv-Uncert*, and *Disadv-Cert*. Finally, we divided our data according to the outcome of the lottery so that we could compare trials in which subjects won or lost money, and then further subdivided the wins depending on the type of lottery the money came from to compare expected and unexpected wins.

Additionally, this study used parametrically weighted predictors to model the effects of ambiguity within the GLM (Buchel et al., 1998). Parametric weights were assigned to each ambiguity condition and the resulting functions were convolved with the canonical hemodynamic response function implemented in BrainVoyager. In order to avoid the partial co-linearity between the main and parametric predictors, the mean of the parametric weights was subtracted from each weight using BrainVoyager. Using de-meaned weights ensured the correlation between parameters was zero, as the predictors were orthogonal to the main boxcar function. We then manually created a design matrix containing both orthogonalized parametric time series so they could be compared within a single GLM in BrainVoyager using the BVQXtools v0.8 (Jochen Weber) toolbox for MatLab. The different functions used to fit the data were based on various levels of uncertainty. First, this study fit a linear function that placed greater weight on higher levels of ambiguity, so that trials with zero ambiguity were associated with a weight of 0 and trials with full ambiguity were associated with a weight of 1. Additionally, the study tested a quadratic “inverted U” function that more strongly weighted intermediate levels of ambiguity. Trials with zero and full ambiguity were assigned a weight of 0, whereas trials with 15% ambiguity were assigned a weight of 0.5. Trials with 33 and 66% ambiguity were assigned a weight of 0.9. Trials with 80% ambiguity were assigned a weight of 0.6. A weight of 1 was not used, as it would correspond to trials with 50% ambiguity not present in the study. This type of weighting resulted in a second order function similar to the Shannon entropy function employed by Bach et al. (2011).

This study controlled for multiple comparisons using the cluster-size thresholding procedure developed by Forman et al. (1995) extended to 3D maps, and implemented in the BrainVoyager Cluster Threshold plug-in (Goebel et al., 2006). An initial map was formed using an uncorrected *p*-value of *p* < 0.005. The minimum cluster size (based on an alpha level of 0.05) was then set by MonteCarlo simulation using 1000 iterations, simulating the stochastic process of image generation. Afterwards, spatial correlations between neighboring voxels were calculated, before voxel intensity thresholds were finally calculated and the corrected map was formed and displayed.

## Results

### Behavioral analysis

**Figure 2:**
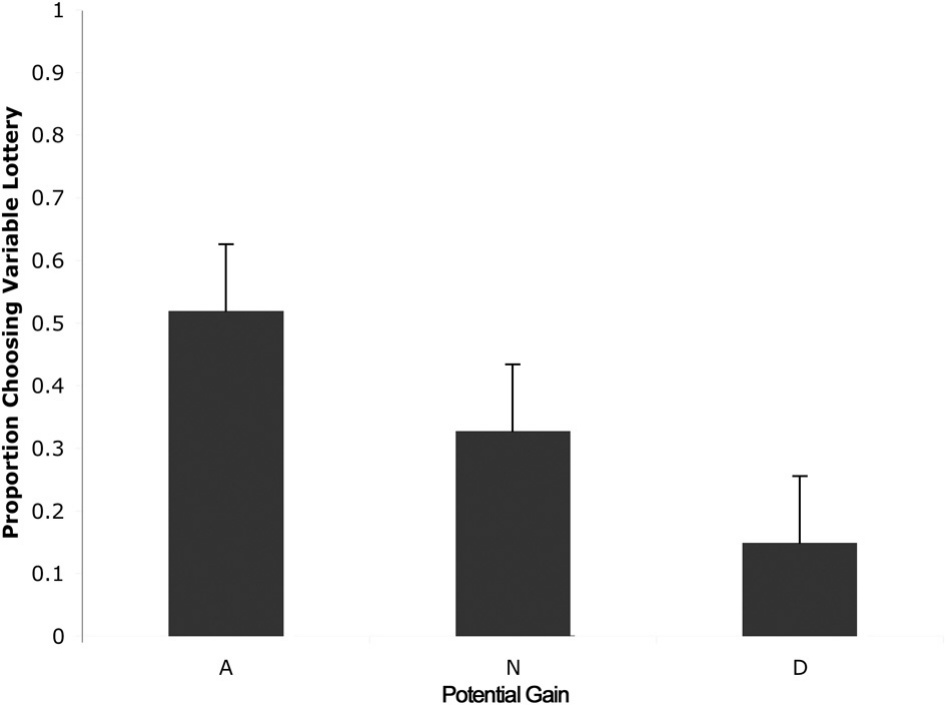
**Behavioral data showing the proportion of ambiguous lotteries chosen across levels of potential monetary gain.** A, Advantageous trials in which potential gain was greater than that of the constant lottery. N, Neutral trials in which potential gain was equal to that of the constant lottery. D, Disadvantageous trials in which potential gain was less than that of the constant lottery Our data suggests that subjects successfully distinguished “good” from “bad” lotteries using the expected payout relative to the constant lottery for choice evaluation.

In order to quantify behavior as a function of uncertainty, choices were defined in terms of the proportion of trials in which subjects chose to play the variable lottery, rather than defining behavior based on the outcome (as monetary gains or losses) of each trial. First, we separated trials according to expected gains, in order to determine whether or not subjects could, in fact, determine a “good” lottery (advantageous trials in which potential gain was greater than the constant lottery) from a “bad” lottery (disadvantageous trials in which potential gain was less than the constant lottery). A One-Way analysis of variance (ANOVA) with factors of expected gain (Advantageous, Neutral and Disadvantageous) revealed a main effect of expected gain [*F*_(2, 39)_ = 15.56; *p* < 0.001]. As shown in Figure 2, *post-hoc* tests using a Games-Howell correction revealed that subjects chose to play the variable lottery when its potential gain was greater than the constant lottery significantly more than when the variable lottery was equal in potential gain to the constant lottery (*p* < 0.05) or when the variable lottery's potential gain was lower compared to the safe lottery (*p* < 0.05). Additionally, subjects decided to play the variable lottery significantly more often when the variable lottery was equal in potential gain to the than when it was lower in potential gain compared to the constant lottery (*p* < 0.05).

We also separated trials according to the level of ambiguity indicated in the variable lottery in order to determine the effect of various levels of ambiguity on choice behavior. A One-Way ANOVA with ambiguity level as a factor (0, 15, 33, 66, 80, 100%) revealed no significant differences in choice behavior across the various different levels of ambiguity. A single sample *t*-test against 0.50 showed an overall effect of avoidance of all types of uncertainty, regardless of whether it was risk or full ambiguity.

**Figure 3:**
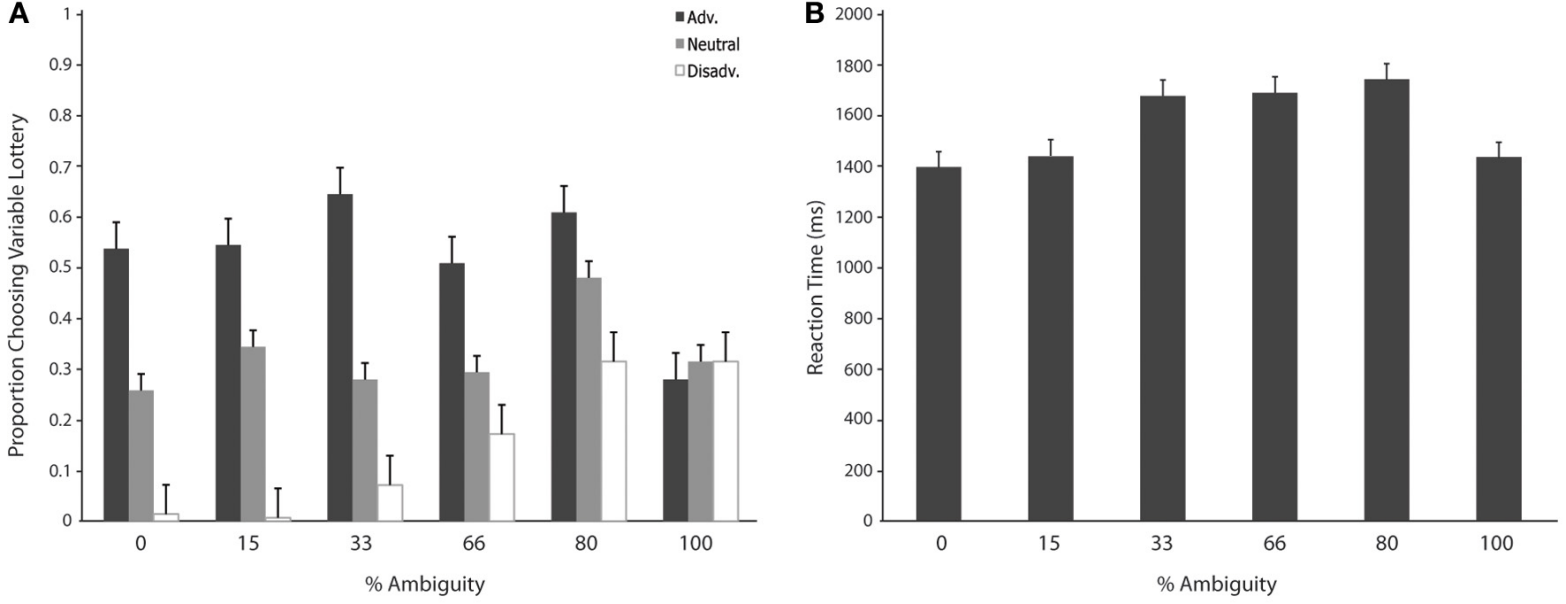
**Choice (A) and reaction time (B) as a function of level of ambiguity. (A)** Proportion of choices of the variable lottery as a function of level of ambiguity and potential gain advantage over the constant lottery. For trials in which it was advantageous to play the variable lottery, subjects demonstrated increased ambiguity aversion (lower probability of choosing the variable lottery) as ambiguity increased. For trials in which it was disadvantageous to play the variable lottery, subjects showed an increase in ambiguity preference possibly caused by the difficulty of distinguishing “good” from “bad” lotteries. For neutral trials, subjects showed ambiguity aversion for the risky option across all levels of ambiguity. **(B)** Reaction times separated by level of ambiguity show a significant difference between low levels of ambiguity (0 and 15%) and intermediate levels of ambiguity (33, 66, and 80%).

We then separated trials according to expected gain and level of ambiguity. A 3 (expected gain) × 6 (ambiguity level) repeated measures ANOVA showed a main effect of expected gain [*F*_(1.23, 15.92)_ = 48.82; *p* < 0.001] and a significant interaction of expected gain and level of ambiguity [*F*_(4.18, 54.31)_ = 13.50; *p* < 0.001]. Maulchy's test indicated that the assumption of sphericity had been violated for expected gain, ambiguity and the expected gain by ambiguity interaction (chi-square = 12.03, 62.05, and 80.43 respectively), which required us to adjust degrees of freedom using a Greenhouse–Geisser estimate of sphericity (epsilon = 0.61, 0.41, and 0.42 respectively). Further analysis via a one-way ANOVA with ambiguity as a factor for advantageous trials revealed significant differences in responses associated with different levels of ambiguity [*F*_(5, 78)_ = 2.96; *p* = 0.02]. As shown in Figure 3, *post-hoc* tests using a Bonferroni correction showed that subjects chose the variable lottery significantly less in trials with 100% ambiguity compared to trials with 30% (*p* = 0.01) and 80% (*p* = 0.04) ambiguity. Similarly, a One-Way ANOVA for disadvantageous trials using ambiguity as a factor also showed significant differences in choice behavior [*F*_(5, 78)_ = 6.43; *p* < 0.001]. Although Levene's test indicated that variances were not homogeneous for this group, both Welch and Brown-Forsythe tests showed significant differences in responses across different ambiguity levels. *Post-hoc* tests using a Games-Howell correction revealed that subjects chose the variable lottery significantly more in trials with 100% ambiguity compared to trials with 0 or 15% ambiguity as shown in Figure 3A. No significant differences were found for neutral trials when broken down by ambiguity levels.

### Reaction times

We first separated trials according to expected gain according to the same criteria used for the choice analyses. A repeated measures analysis of variance (ANOVA) with factors of expected gain (Advantageous, Neutral and Disadvantageous) revealed no significant differences in response times. Additionally, we separated trials according to whether subjects chose to play the variable or safe lottery, but found no significant differences in response time. Finally, we separated trials according to the level of ambiguity indicated in the variable lottery in order to determine the effect of various levels of ambiguity on choice behavior. A repeated measures ANOVA with ambiguity level as a factor (0, 15, 33, 66, 80, 100%) revealed a main effect of reaction times as a function of the various different levels of ambiguity. [*F*_(1, 5)_ = 7.58; *p* < 0.001]. As shown in Figure 5, *post-hoc* tests using a Bonferroni correction (*p* = 0.05) showed that subjects' reaction times were faster for trials with 0% ambiguity compared to trials 33, 66, and 80% ambiguity. Reaction times were also significantly lower for trials with 15% ambiguity compared to trials with 33, 66, and 80% ambiguity.

### Comparison between conditions within the whole-brain analysis

#### Uncertain vs. certain choices

To examine the overall pattern of neural activity associated with uncertainty, we combined all trials in which subjects chose to play the variable lottery, regardless of outcome and contrasted that against trials in which subjects chose to play the safe lottery (*Uncertain* > *Certain*). Using this contrast, we observed increased activity associated with uncertain choices, including tracking of anticipated value, bilaterally in the fronto–parietal–striatal decision making network, specifically within the DLPFC and frontal pole, parietal cortex in the intraparietal sulcus, and putamen. Additionally, we observed increased activity in the insula. All these regions have been associated with judgment under uncertainty in previous studies (Brand et al., 2006) which may reflect the increased cognitive demand of trying to compute anticipated reward that is not necessary when choosing the safe lottery, as the expected and experienced values are the same. There was higher activity for certain choices in the OFC and ventral striatum as shown in Table 1. Greater activity in both of these regions was not surprising as these areas are associated with processing the value of options, as expressed in the uncertain lottery in which the value was not anticipated but known.

**Table 1:** **Areas of activation associated with uncertainty**.

**Contrast**	**Region of activation**	**Number of voxels**	***BA***	***x***	***y***	***z***
Uncertain > certain	DLPFC–R	96	46	39	20	37
	DMPFC–Bi	2256	8	2	19	50
	Frontal eye fields–R	1027	6	39	7	48
	Frontal pole–R	132	10	33	61	11
	Inferior occipital cortex–R	58	19	46	−73	−24
	Insula–L	65	13	−32	21	4
	Insula–R	1312	13	34	20	5
	Middle temporal gyrus–L	91	21	−60	−32	−6
	Middle temporal gyrus–R	484	21	61	−31	−3
	Occipital cortex–L	625	18	−14	−87	−19
	Occipital cortex–R	156	18	14	−96	1
	Occipitotemporal junction–R	67	37	51	−49	9
	Posterior cingulate–L	391	33	4	−29	25
	Putamen–L	56	–	−27	2	−4
	Superior parietal cortex–L	2195	7	−46	−57	44
	Superior parietal Cortex–R	4417	7	33	−66	48
	Supramarginal gyrus–R	271	39	47	−53	33
Certain> uncertain	Cuneus–L	345	17	−9	−61	15
	Superior temporal gyrus–R	52	38	47	9	−9
	Ventral striatum–Bi	380	–	−1	11	2
	VMPFC–L	190	32	−3	42	−4

BA, Brodmann areas; x,y,z, Talairach coordinates (Talairach and Tournoux, 1998) of central voxel in activated cluster. Cluster size threshold based on uncorrected voxelwise p < 0.005 and cluster size alpha < 0.05.

#### Ambiguity vs. risk

**Figure 4:**
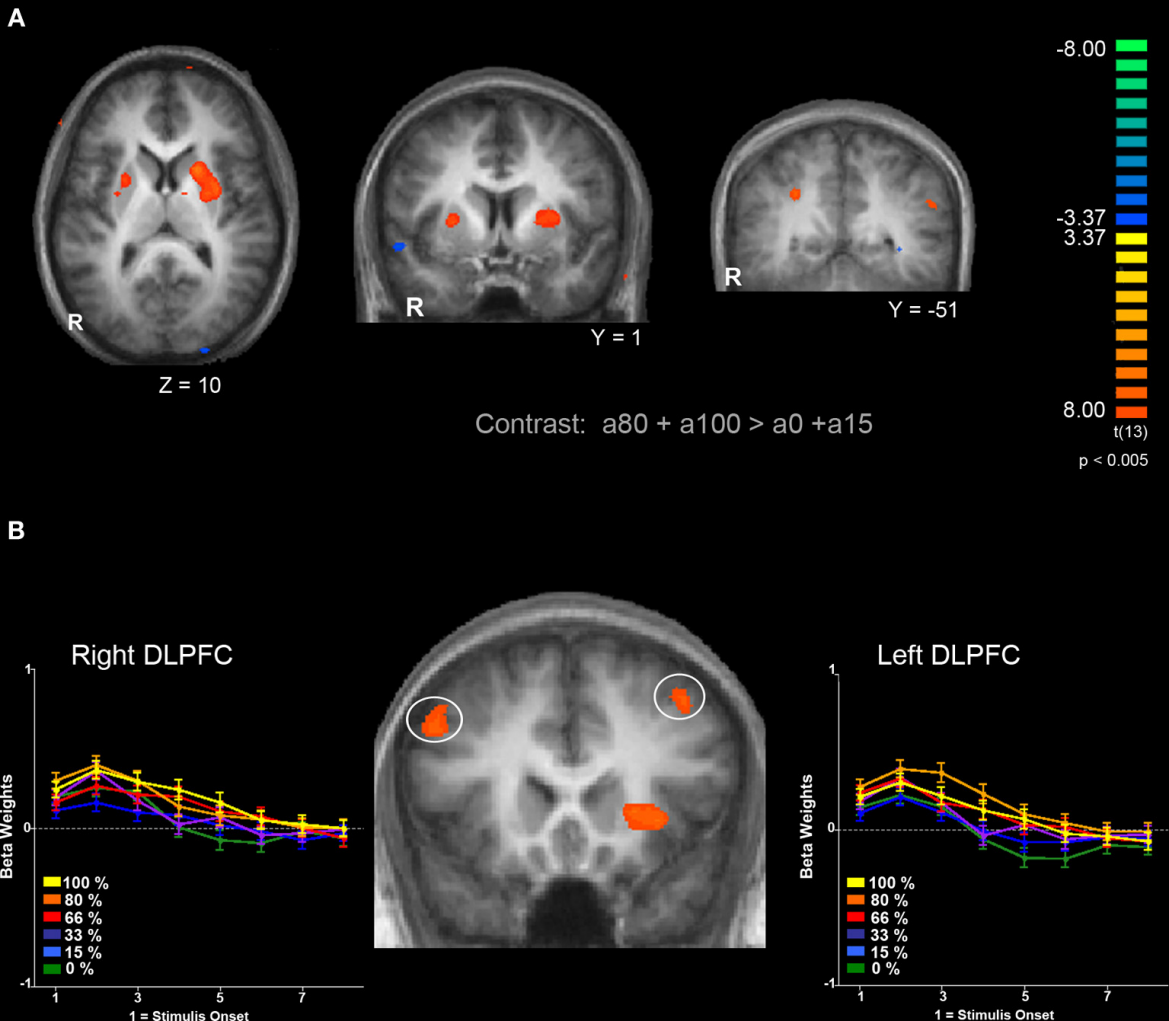
**Comparison of BOLD responses associated with ambiguous decisions (a85 and a100) compared to risky decisions (a0 and a15). (A)** Left and middle panels show bilateral putamen activation. Right panel shows bilateral activation of the parietal lobe within the intraparietal sulcus. **(B)** Bilateral activation in the DLPFC and response to different levels of ambiguity. Functionally defined ROIs in both left (*x* = −45, *y* = 17, *z* = 37) and right (*x* = 34, *y* = 21 *z* = 47) hemispheres of the DLPFC show a similar pattern of activation; a greater response for trials with full ambiguity compared to trials with no ambiguity, along with increased response to partially ambiguous trials. Functional maps are overlaid on a T1-weighted average of all 15 participants' anatomicals. Cluster size threshold based on uncorrected voxelwise *p* < 0.005 and cluster size alpha < 0.05 as indicated in the methods section.

Next, we were interested in exploring differences in neural activity associated with specific types of uncertainty, categorically defined as risk or ambiguity in previous papers. First, we compared trials with 100% ambiguity (a100 trials) against trials with 0% ambiguity (a0 trials) resulting in our *a100 > a0* contrast that matched the Huettel et al. (2006) ambiguity vs. risk contrast and the Bach et al. (2009) ignorance vs. risk comparison. To increase statistical power, we also expanded this contrast to include more trials at either range of the uncertainty spectrum by comparing trials with high levels of ambiguity (a80 + a100) to trials with low levels of ambiguity (a0 + a15) resulting in our *a100 + a80 > a0 + a15* contrast. Overall, the pattern we found in both the *a100 > a0* and *a100 + a80 > a0 + a15* contrasts were similar, but the latter contrast resulted in more robust and symmetrical patterns of activity, likely due to the inclusion of a larger number of trials. Of particular note, we observed increased activity associated with high levels of ambiguity across the fronto–parietal–striatal network, including the DLPFC, frontal pole, intraparietal sulcus, putamen, and head of the caudate. The region of the DLPFC recruited was in a region of middle frontal gyrus typically considered to be Brodman's area 9/46v (Badre and D'Esposito, 2009). Additionally, we observed increased activity bilaterally in the hippocampus associated with risk. See Table 2 for a full list of activated regions, and Figure 4 for an illustration. In order to further examine DLPFC recruitment as a function of increasing degrees of ambiguity we plotted beta values across time within a functionally defined Region of Interest (ROI) taken from our *a100 + a80 > a0 + a15* contrast, shown in Figure 4B. Overall there was greater activity for both fully and partially ambiguous trials compared to risk trials.

**Table 2:** **Areas of activation associated with Ambiguity**.

**Contrast**	**Region of activation**	**Number of voxels**	***BA***	***x***	***y***	***z***
a100 > a0	DLPFC-L	100	46	−45	20	37
	Inferior temporal gyrus-L	395	20,21	−57	−29	−16
	Lingual/fusiform gyrus-L	2242	19,37	−41	−53	−17
	Middle temporal gyrus-L	176	21	−50	−25	−9
	Occipital cortex-L	1318	17,18,19	−26	−77	−14
	Occipital cortex-R	956	17,18	25	−68	−5
	Post-central gyrus-R	1127	2,40	44	−19	23
	Premotor cortex-Bi	444	6	0	−3	50
	Putamen/caudate-R	495	–	21	14	5
	Putamen-R	389	–	28	3	12
	Superior parietal cortex-L	256	7	−18	−33	59
a100 + a80 > a0 + a15	Anterior cingulate gyrus-R	27	24	9	23	35
	DLPFC-L	317	46	−45	17	37
	DLPFC-R	495	9,46	34	21	47
	Frontal pole-L	34	10	−26	51	24
	Frontal pole-R	531	10	41	48	14
	Inferior temporal gyrus-R	743	20,21	53	−25	−12
	OFC	107	11	18	61	−2
	IPS-L	92	39	−51	−50	27
	IPS-R	294	39	27	−50	33
	Posterior parietal cortex-L	333	7	−13	−77	36
	Premotor cortex-R	78	6	51	9	26
	Putamen/caudate/insula-L	1026	13	−26	2	8
	Putamen/caudate/insula-R	3021	13	24	7	8
	Superior parietal lobe-R	334	7	2	−80	35
a0 + a15> a100 + a80	Hippocampus-L	692	–	−26	−30	−10
	Hippocampus-R	98	–	27	−33	−4
	Middle temporal gyrus-L	211	21	−52	1	−5
100 > 15	Anterior cingulate-R	60	23	8	−22	33
	DLPFC-R	248	46	36	47	12
	DLPFC-R	98	9,46	29	20	45
	Frontal pole-L	99	10	−21	54	2
	Frontal pole-L	52	10	−30	56	6
	Middle temporal gyrus-L	61	21	−58	−14	3
	Motor cortex-R	634	4	57	−16	12
	Motor cortex-R	177	4	54	1	11
	Parietal cortex-L	219	7	−65	−44	22
	Putamen-R	164	–	28	0	10
	Putamen-R	108	–	22	12	5
	Superior parietal cortex-L	635	7	−15	−52	65
	Superior parietal cortex-L	63	7	−13	−50	56
15 > 100	Hippocampus-R	126	–	35	−32	−3
	Occipital cortex-R	333	18,19	8	−94	−5
	Occipital cortex-R	58	18	29	−92	−9
	VMPFC-L	145	47	−6	24	−9
33 + 66 > 100	Angular gyrus-R	92	39	56	−43	0
	DLPFC-R	149	9	31	3	36
	OFC-R	331	11	7	58	5
	Parahippocampal gyrus-L	133	36	−32	−21	−30
	Posterior cingulate-R	607	30	18	−51	25
	Premotor-R	223	6	29	1	67
	Superior frontal gyrus-L	115	9	−18	47	47
100 > 33 + 66	DMPFC-L	943	32	−8	36	13
	Inferior temporal gyrus-R	66	21	61	−7	−28
	Motor cortex-R	1249	4	−1	−12	52
	Motor cortex-L	144	4	2	−22	63
	Superior parietal cortex-R	263	7	5	−43	67
	Superior parietal cortex-L	149	7	−20	−49	59

BA, Broadmans areas; x,y,z, Talairach coordinates (Talairach and Tournoux, 1998) of central voxel in activated cluster. Cluster size threshold based on uncorrected voxelwise p < 0.005 and cluster size alpha <0.05.

It is important to note that the 0% ambiguity or risk condition presents a special case in that it can be considered a one-stage gamble, as opposed to two, given that uncertainty is only associated with the outcome. We therefore ran a series of analyses examining levels of ambiguity while excluding this condition. First, we compared trials with high ambiguity (a100) against trials with low ambiguity (a15), to examine effects of uncertainty in the absence of risk, and found similar patterns of activation as seen in the contrasts that included risk (i.e., a100 > a0, and a100 + a80 > a0 + a15) presented above. Mainly, we still see increased activity in the DLPFC, frontal pole, parietal cortex and putamen as reported in the contrasts that include risk as shown in Table 2.

With the concerns of excluding the 0% ambiguity condition due to a potential confound, we also compared trials with intermediate amounts of partial ambiguity against trials with complete ambiguity (a33+a66 > a100), which was analogous to Bach and colleagues (2009, 2011) ambiguity vs. ignorance contrast. We found small regions of increased activity in superior portion of the DLPFC in the superior frontal gyrus and OFC associated with partial ambiguity and greater activity in the superior parietal cortex for complete ambiguity.

### Parametric whole-brain analysis of ambiguity

Table 3 shows a complete list of activated regions associated with each parametric model. Each parametric regressor is described in the Methods, above. We first examined regions of activity predicted by the quadratic inverted-U model that put a greater emphasis on intermediate levels of ambiguity. We found areas of increased activation bilaterally in regions of the fronto–parietal–striatal decision making system including the DLPFC, frontal pole, intraparietal sulcus and posterior parietal cortex, and putamen and caudate. The region of the DLPFC recruited include parts of both the middle and superior frontal gyri typically considered to be Brodman's area 9/46v, and 9/46d (Badre and D'Esposito, 2009). This pattern of results in the frontal cortex is consistent with claims that activity associated with ambiguity processing in the DLPFC is greatest when only intermediate levels of ambiguity are weighted (Bach et al., 2009). We also found a region of the OFC negatively related to the quadratic regressor, indicating overall higher activity for full risk and/or full ambiguity in contrast with partial ambiguity.

**Table 3:** **Areas of activation resulting from parametric manipulation**.

**Model**	**Region of activation**	**Number of voxels**	***BA***	***x***	***y***	***z***
Quadratic	Body/tail of caudate-R	2588	–	17	−14	20
	DLPFC-R	1744	9,46	46	24	34
	DLPFC-R	1486	9	30	41	43
	Frontal pole-R	164	10	9	60	3
	Middle temporal gyrus-R	209	37	58	−56	−6
	Parietal cortex-L	1194	39	−41	−45	33
	Parietal cortex-R	9419	7,39,40	19	−64	36
	Pre-central gyrus-L	1119	4	−27	−7	64
	Pre-central gyrus-R	4440	4,6	29	0	54
	Pre-central gyrus-R	183	4	15	−3	57
	Premotor cortex-L	491	6	−32	1	33
	Putamen-L	643	–	−23	−1	17
	Putamen-L	315	–	−28	−4	2
	Putamen-R	1882	–	27	−8	2
	Superior parietal cortex-L	1398	7	−17	−71	55
	VLPFC-R	256	45	40	30	8
	**Anterior cingulate gyrus-L**	**204**	**24**	**−5**	**33**	**21**
	**OFC-L**	**467**	**24,32**	**−8**	**37**	**12**
Linear	DLPFC-L	246	46	−46	19	37
	DLPFC-R	283	9,46	33	21	47
	Inferior temporal gyrus-L	776	20,21	−58	−28	−16
	Insula-L	230	13	−31	12	6
	Parietal cortex-L	179	7	−13	−77	37
	Precuneus-L	257	17,18,31	−25	−70	26
	Putamen/insula-R	2271	13	24	11	11
	Superior parietal cortex-L	174	40	−26	−53	44
	**OFC-L**	**33**	**11**	**−2**	**36**	**−10**
	**Parahippocampal gyrus-L**	**216**	**36**	**−26**	**−31**	**−11**
Quadratic>Linear	Angular gyrus-R	60	39	45	−55	7
	DLPFC-R	537	9,46	18	27	57
	DLPFC-R	122	9	13	39	51
	Middle temporal gyrus-R	95	21	53	−46	−1
	Motor cortex-R	89	4	28	−2	64
	Occipital cortex-R	122	17,18	20	−96	15
	Posterior parietal cortex-R	937	7	16	−80	45
	Posterior cingulate gyurs-L	83	23	−10	−27	33
	Posterior cingulate gyurs-R	124	30	15	−53	25
	VMPFC-R	360	11	5	55	3
Linear > Quadratic	Anterior cingulate gyrus-L	150	24	−8	32	11
	Motor cortex-L	95	4	−3	−10	56
	Parahippocampal gyrus-L	173	36	−23	−33	−13
	Superior parietal cortex-L	117	7	3	−42	63

BA, Brodmann areas; x,y,z, Talairach coordinates (Talairach and Tournoux, 1998) of central voxel in activated cluster. Cluster size threshold based on uncorrected voxelwise p < 0.005 and cluster size alpha <0.05. Bold items indicate negative t-values.

**Figure 5:**
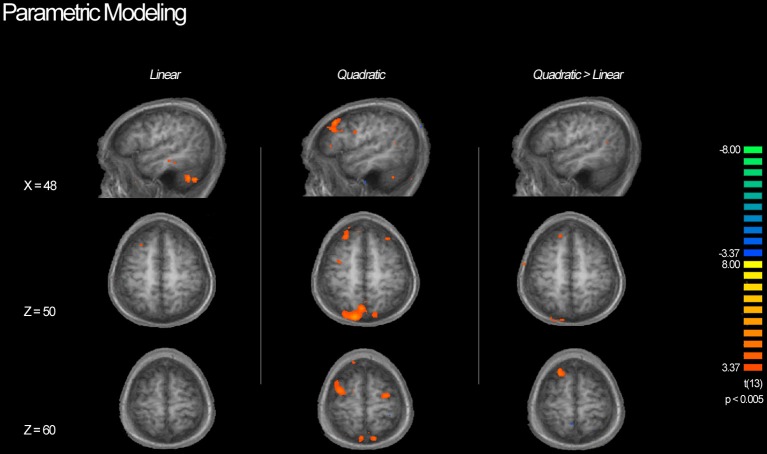
**BOLD activity associated with parametric models**. Left column shows areas of activity associated with a linear parametric regressor weighting trials by the proportion of ambiguity. Middle column shows areas of activity associated with an “inverted U” quadratic function in that most strongly weighted trials with intermediate levels of ambiguity. Right column shows areas of activity associated with the inverted U model when the contributions of the linear model are controlled for. Although we see activity in the DLPFC associated with both the linear and quadratic models, the inverted U model recruits additional superior and anterior regions as well as posterior parietal cortex, suggesting that decisions associated with intermediate levels of ambiguity require more cognitive processing throughout the frontoparietal decision making network.

We then examined the linear model in which ambiguity increased in a linear fashion in order to explore if activity in any regions was related to absolute magnitude of ambiguity. In this analysis risk trials (trials with no ambiguity) had a zero weight, whereas trials with complete ambiguity were given the maximum weight of one (Figure 5, left column), intermediate trials were weighted according to the percent of the dial obscured by the occlusion. Overall regions recruited in this analysis were much smaller, but included many of the same regions of the fronto–parietal–striatal network that were sensitive to the quadratic function (Table 3, middle section). One small region of the OFC and one region of the parahippocampal gyrus were negatively related to the linear function, indicating less recruitment for higher levels of ambiguity.

Finally, we directly compared both the “inverted-U” quadratic model against the linear model. We found greater activity for the quadratic model in relatively superior and anterior regions of the DLPFC in the superior frontal gyrus (BA9/46d; Badre and D'Esposito, 2009). In addition, there was greater medial parietal and angular gyrus recruitment for the quadratic model than the linear model. Alternately, regions of the superior parietal cortex and anterior cingulate were more active for the linear model.

### Expected gain

We examined neural responses associated with relatively “good” vs. “bad” decisions by grouping trials according to our interpretation of expected gain. Here, we compared advantageous trials in which the expected gain associated with the variable lottery was greater than that of the safe, or constant, lottery against disadvantageous trials in which the expected gain of the variable lottery was lower than that of the constant lottery *(Adv > Disadv)*. For advantageous trials we observed greater activation in some regions of the fronto–parietal–striatal system, in particularly the DLPFC and intraparietal sulcus. Additionally, there was bilateral activity in the insula. Disadvantageous trials were associated with increased activity in the left OFC.

**Figure 6:**
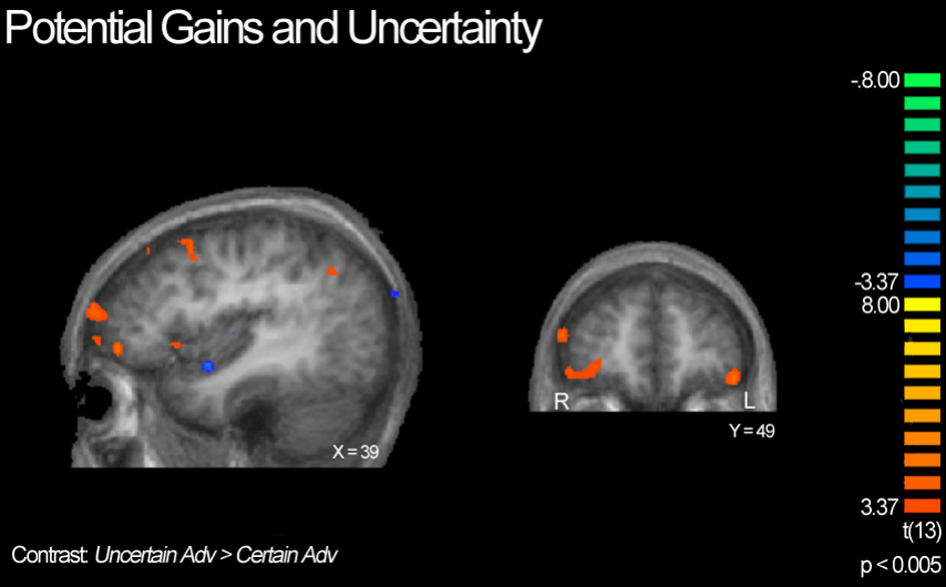
**BOLD responses showing interaction of potential gain and uncertainty**. Neural activity associated with subject's choice of the uncertain lottery over the certain lottery in situations where the uncertain lottery was associated with a *greater* potential monetary gain than the certain lottery based on the *Uncertain Adv > Certain Adv* contrast. Left panel shows activity in the orbitofrontal cortex, DLPFC, frontal pole, insula and parietal cortex; right shows bilateral activity in the orbitofrontal region. Here we demonstrate how uncertainty continuously modulates activity in regions previously implicated in only the valuation of stimuli/options.

Next, we compared advantageous and disadvantageous trials accounting for type of lottery played, certain or uncertain. For example, we compared trials in which subjects chose the variable lottery over the constant lottery for only advantageous trials (*Uncertain Adv > Certain Adv*). Based on this contrast, we observed right lateralized activation throughout the DLPFC and frontal pole, and OFC, as illustrated in Figure 6. Additionally, we observed increased activation in the posterior cingulate and left tail of the caudate. In contrast, the Certain Advantageous trials led to greater activity than uncertain bilaterally in the motor cortex, left hippocampus and right parahippocampal gyrus. Finally, we examined disadvantageous trials in which subjects chose the variable lottery over the constant lottery (*Uncertain Disadv > Certain Disadv*). This led to a very different pattern of recruitment than when advantageous trials were examined: there was widespread activation throughout the bilateral anterior insula, frontal poles, and intraparietal sulcus. See Table 4 for a complete list of activated regions.

**Table 4:** **Areas of activation associated with expected gains**.

**Contrast**	**Region of activation**	**Number of voxels**	***BA***	***x***	***y***	***z***
Adv. > Disadv.	DLPFC-L	215	45	−47	16	17
	Inferor temporal gyrus-L	171	21	−46	−67	−11
	Insula-L	305	13	−36	20	6
	Insula-R	768	13	37	21	4
	Middle temporal gyrus-L	1772	21	−56	−42	3
	Superior parietal cortex-L	2759	7	−34	−63	55
	Superior parietal cortex-R	2350	7	36	−63	49
	Supplementary motor area-L	371	6	−52	5	48
	Supplementary motoro area-R	658	6	43	9	50
Disadv. > Adv.	Fusiform gyrus-L	849	20	−31	−21	−22
	Occipitoparietal fissure-L	218	31	−18	−59	22
	VMPFC-L	935	32	−13	31	10
Uncertain disadv. > Certain disadv.	Frontal pole-L	262	10	−32	57	11
	Frontal pole-R	2561	10	41	54	14
	Frontal pole-R	512	10	14	66	10
	Insula-L	272	13	−27	21	−4
	Insula-L	711	13	−42	14	6
	Insula-R	2704	13	32	19	2
	Occipital cortex-L	322	19	−38	−74	−14
	SMA-R	484	6	15	13	62
	SMA-R	372	6	4	3	67
	Superior parietal-L	257	7	−30	−58	41
	Superior parietal-L	253	7	−34	−62	53
	Superior parietal-R	2336	7,40	26	−67	51
Uncertain adv. > Certain adv.	DLPFC-R	302	9	40	12	50
	DLPFC-R	525	9	24	18	59
	DLPFC-R	228	9	23	34	56
	DMPFC-R	501	8	3	35	42
	Frontal pole-L	181	10	−26	68	2
	Frontal pole-R	2521	10	34	59	21
	Middle temporal gyrus-L	211	21	−58	−29	−5
	Occipital lobe-R	220	18	25	−89	−13
	Occipital-parietal junction-R	783	39	45	−59	32
	OFC-L	386	11	−38	47	−2
	OFC-R	1185	11	39	51	4
	OFC-R	806	11	18	67	6
	Posterior cingulate-Bi	1700	23	1	−28	32
	Tail of caudate-L	232	–	−19	−23	26
Certain adv. > Uncertain adv.	Hippocampus-R	327	−	31	−10	−15
	Parahippocampal gyrus-L	236	36	−29	−34	−18
	Pre-central gyrus-L	422	4	−61	−24	26
	Pre-central gyrus-R	399	4	54	−10	22
	Superior parietal-L	607	2	−50	−31	53

BA, Brodmann areas; x,y,z, Talairach coordinates (Talairach and Tournoux, 1998) of central voxel in activated cluster. Cluster size threshold based on uncorrected voxelwise p < 0.005 and cluster size alpha < 0.05.

### Wins vs. losses

We examined the general pattern of activation associated with either winning or losing money; however, one must take care in interpreting results given that our analysis window included both evaluation and reward/outcome events. Here, our main contrast compared trials in which subjects gained money, regardless if they chose to play the uncertain or safe lottery, against trials in which subjects lost money. We observed increased activity for wins across frontal and striatal regions known to be sensitive to reward, including the OFC, DMPFC, DLPFC, ventral striatum, body of the caudate, medial temporal lobe, regions of the medial parietal cortex including the posterior cingulate, precuneus, and cuneus, as well as the left intraparietal sulcus. Additionally, we found activity associated with losing money in the right insula, right frontal pole, and right superior parietal cortex.

Next, we looked more closely at wins, and separated them according to reward expectancy so that trials in which the outcome was uncertain (unexpected wins) were contrasted against trials in which the outcome was certain (expected wins). This contrast revealed that unexpected wins recruited similar regions to wins vs. losses overall, including intraparietal sulcus, posterior cingulate, OFC, DLPFC and the frontal pole as listed in Table 5. One salient difference was the strong recruitment of the putamen and bilateral insula for unexpected wins. The only region more active for expected than unexpected wins was the OFC. Because this task did not allow for a condition in which the subjects were ever faced with a certain loss, we did not look at differences between expected and unexpected losses.

**Table 5:** **Areas of activation associated with winning money**.

**Contrast**	**Region of activation**	**Number of voxels**	***BA***	***x***	***y***	***z***
Wins > Losses	Angular gyrus-R	562	40	49	−33	25
	Cuneus-R	166	19	10	−88	30
	DLPFC-L	1921	46	−26	23	36
	DMPFC-R	682	8	4	35	38
	Frontal pole-L	354	10	−8	59	21
	Fusiform gyrus-L	1343	37	−30	−48	−16
	Hippocampus-R	1930	–	32	−11	−14
	Insula-L	334	13	−39	−8	6
	Occipital cortes-L	118	17	−26	−81	10
	Occipital cortex-L	359	18	−6	−70	−7
	Occipital cortex-R	3965	17,18,19	19	−56	−3
	Occipital cortex-R	104	17	5	−88	3
	Occipitoparietal junction-R	1453	22,39	44	−68	10
	OFC-L	217	11	−28	38	−1
	Parahippocampal gyrus-L	263	–	−23	−15	−7
	Parahippocampal gyrus-R	281	–	30	−29	−15
	Post-central gyrus-R	168	1,2	62	−26	26
	Posterior caudate-R	1558	–	22	−20	31
	Posterior cingulate gyrus-R	1386	23,31	11	−37	47
	Posterior cingulate/precuneus-L	5643	23,30,31	−15	−58	14
	Pre-central gyrus-R	259	4	55	2	11
	Superior parietal cortex-L	8150	2,7,40	−23	−36	51
	Superior parietal cortex-R	402	7	16	−35	62
	Superior temporal gyrus-L	3315	40,42	−56	−25	16
	Superior temporal Gyrus-R	540	41,42	51	−18	9
	Ventral striatum-Bi	1126	–	0	12	−1
	VMPFC-L	182	32	−11	48	9
*Losses > Wins*	Frontal eye fields-R	769	8	4	20	55
	Frontal pole-R	6227	10	30	61	12
	Inferior frontal gyrus-R	568	45	42	19	7
	Middle temporal gyrus-R	417	21	58	−31	−2
	Superior parietal cortex-R	2977	7,40	49	−62	42
	Supplementary motor area-R	146	6	38	7	54
Unexpected wins > Expected wins	DLPFC-L	968	9,46	−39	45	28
	DLPFC-R	476	9	26	33	53
	Frontal eye fields-R	1367	8	14	15	65
	Frontal pole-R	1508	10	34	54	28
	OFC-L	881	11	−34	47	4
	Posterior caudate-L	2572	–	−19	−18	21
	Posterior cingulate gyrus-Bi	4131	23,31	−1	−29	28
	Premotor cortex/DLPFC-R	4667	6,46	40	8	41
	Premotor cortex-L	731	6	−47	6	52
	Putamen/insula-L	850	13	−28	−3	−3
	Putamen/insula-R	3032	13	30	14	4
	Superior colliculi-Bi	340	–	0	−33	−2
	Superior parietal cortex-Bi	8985	7,40	8	−64	44
Expected wins > Unexpected wins	VMPFC-L	812	32	−4	30	−1

BA, Brodmann areas; x,y,z, Talairach coordinates (Talairach and Tournoux, 1998) of central voxel in activated cluster. Cluster size threshold based on uncorrected voxelwise p < 0.005 and cluster size alpha < 0.05.

## Discussion

The present study demonstrated changes in regional brain activation as a function of varying levels of uncertainty. First, we identified a set of brain regions that showed an increase in activation in response to increased uncertainty compared to situations of low uncertainty. We demonstrated that both risk and ambiguity modulate activation in a subset of regions generally activated by uncertain decision making: the DLPFC, parietal cortex, striatum and anterior insula. More importantly, we demonstrated that ambiguity processing in regions of the prefrontal cortex does not necessarily scale linearly with the level of ambiguity, but rather the inherent difficulty of the decision. We found evidence to suggest that while activity in the middle frontal gyrus region of the DLPFC is sufficient for the successful processing of contextual information during uncertain decision making, recruitment of superior and anterior regions of the DLPFC is maximal during conditions of partial ambiguity.

### Risk vs. ambiguity

We were interested in exploring whether ambiguity is a continuously quantifiable variable representing uncertainty on action-outcome associations separate from risk. We found a set of activated regions across all levels of uncertainty, reinforcing the idea that both risk and ambiguity recruit a shared network that includes the DLPFC, striatum, intraparietal sulcus, and insula. In addition we observed a similar pattern of greater activity for ambiguity in the fronto–parietal–striatal system and the insula when we compared various levels of ambiguity and excluded the risk condition. Smith et al. (2002) found distinct patterns of activity for risk and ambiguity: ambiguity modulated activity in the OFC more than risk did, and recruited regions of the lateral prefrontal cortex that were not recruited for risk. Later studies did not find as marked differences in activation patterns across the brain for risk and ambiguity. Huettel and colleagues (2006) found that risk and ambiguity share many of the same neural substrates overall, but that regions of the prefrontal cortex, DLPFC (−41, 18, 26) especially, were highly correlated with individual ambiguity preferences whereas regions of the posterior parietal cortex and intraparietal sulcus were correlated with individual risk preferences. In our study we did not examine individual differences in risk preference, and in fact excluded potential subjects with extreme ambiguity aversion or preference. Instead we examined degree of partial ambiguity and found that the regions of DLPFC and parietal lobe identified by Hsu et al. (2005) and Huettel et al. (2006), showed increased activity for trials with partial ambiguity throughout the fronto–parietal–striatal network. Thus, our results add to these earlier studies by providing evidence that these regions are active for various levels of uncertainty.

### Ambiguity processing in PFC

Through our parametric analyses, we observed increased activation in various regions associated with ambiguity processing. We found overall higher activity in the DLPFC, intraparietal sulcus, and dorsal striatum for both a linear increasing and inverted U-shaped quadratic functions, but with greater recruitment by the inverted U-shaped function, indicating that these regions were particularly sensitive to partial ambiguity. Overall, these results are consistent with increased activity in the DLPFC associated with ambiguity processing reported previously across different studies (Hsu et al., 2005; Rustichini et al., 2005; Huettel et al., 2006; Levy et al., 2010). In addition, this pattern of results is similar to that (with the exception of the DLPFC) found by Bach and colleagues (2011) using a similar second-order function to model uncertainty.

The increased activity in the DLPFC for intermediate ambiguity compared to situations with no ambiguity (risk) or full ambiguity (ignorance) is consistent with the hypothesis that neural computational demands change as a function of the level of ambiguity. The inverted-U pattern of activity may reflect not just a search for information (context), but rather a search for *useful* information which is greatest in trials that contain intermediate levels of ambiguity, as suggested by Bach and colleagues (2009). We extended Bach et al.'s (2009) findings by examining more discrete levels of uncertainty, ranging from no ambiguity to complete ambiguity, and by examining ambiguity within a decision making context rather than in a passive conditioning task. We propose that for risky decisions, one does not need to search for context, as all necessary information regarding possible outcomes is readily available. Conversely, it may be difficult or impossible to search for context during fully ambiguous decisions because of the complete lack of information regarding possible outcomes. It is only during situations involving partial ambiguity where it may be beneficial to devote additional cognitive resources to contextual search, which can be done by evaluating what Huettel and colleagues (2006) call the “multiplicity of all possible interpretations” for each option. This interpretation of DLPFC function accounts for results in previous studies showing activity in the posterior inferior frontal gyrus during outcome prediction when contextual cues implied uncertainty across various tasks (Huettel et al., 2005; Li et al., 2006) as well as our results.

In this light, it is interesting to note that activity in relatively superior and anterior regions of the PFC was better represented by our quadratic function in which partially ambiguous trials were most strongly weighted. Anterior regions of the PFC were also recruited for trials in which the advantageous choice was uncertain, and for unexpected wins. One popular framework of frontal lobe function suggests that the prefrontal cortex is organized in a hierarchical manner in which different regions support various aspects of cognitive control (Koechlin and Summerfield, 2007; Badre and D'Esposito, 2009). In this framework, contextual control (maintenance of task rules and structure) is associated with posterior regions, whereas episodic control (maintenance of information in a temporal domain) is associated with activity in more anterior regions. In other words, as task demands increase or tasks become more complex, regions of the prefrontal cortex can be recruited in a posterior to anterior fashion to provide the necessary neural processing. For example, Koechlin and colleagues compared task cuing, which was presumed to be primarily contextual, and response cuing, which was presumed to be primarily episodic (Koechlin et al., 2003). Greater activation to the contextual task cuing was observed in lateral prefrontal cortex regions anterior to regions of activity shared in both tasks. Similarly, Brass and von Cramon (2004), investigated regions of the PFC necessary for contextual processing and found activation in the lateral parts of the prefrontal cortex.

Our observed patterns of activation are consistent with trials in which additional cognitive control is required, and is consistent with emerging views of the frontopolar cortex (Koechlin and Summerfield, 2007; Badre, 2008; Botvinick, 2008). As argued above, the partial ambiguity trials benefit the most from recruitment of additional cognitive processes, as also evidenced by the observed increase in reaction times. Partial ambiguity requires not only contextual control, thought to require DLPFC, as one integrates various decision variables from the current stimulus, but also episodic control, thought to require the frontal pole, as one integrates outcomes and history of reward from previous trials in an attempt to make the best choice given the limited amount of information available presented in the current trial. Finally, this idea is supported by a recent finding by Burke and colleagues (2013) showing that while risk and cognitive effort are calculated separately in the brain, the frontal poles are involved in linking effort and risk during decision making (Burke et al., 2013). It is important to note that our parametric results make only relative comparisons between the two models tested and may not provide a complete account of the nature of processing in the discussed neural regions. In order to establish mechanistic roles for each neural region requires further study.

### Ambiguity, loss, and the insula

Our results are consistent with previous neuroimaging studies that found that the anterior insula is recruited for decision making under conditions of uncertainty, including both risk and ambiguity (Paulus et al., 2003; Sanfey et al., 2003; Huettel et al., 2005; Kuhnen and Knutson, 2005). We observed bilateral insula activation when we collapsed trials across all levels of uncertainty and compared trials in which subjects chose to play the variable lottery vs. the constant lottery. This pattern of results matches that found by Paulus and colleagues (2003) who also found increased activity in the anterior insula when subjects chose to place safe bets as opposed to risky ones.

Insula activation occurs in a wide variety of task conditions. There is, however, an emerging consensus that insula activation is frequently associated with reactions to aversive stimuli or situations (Hester et al., 2010; Mohr et al., 2010), in particular loss (Chua et al., 2009; Mohr et al., 2010) and learning from punishment (Samanez-Larkin et al., 2008; Hester et al., 2010). Our results show that the insula was recruited when subjects chose the variable lottery on disadvantageous trials, and for unexpected wins. Finally, we found the insula was more active for losses than for wins. These lines of evidence, as well as our previous observation that activity in the anterior insula is present as uncertainty increases, suggests that activity in the anterior insula may not reflect uncertainty processing or other similar decision making variables. Rather, insular activation is modulated by the potential of negative or adverse outcomes and is consistent with the theory that insula activity reflects cognitive and emotional processes linked to the anticipation of and experience of aversive situations (Chua et al., 1999; Sawamoto et al., 2000; Ploghaus et al., 2001), such as not being able to predict whether a future outcome will be rewarding or punishing, as observed for unexpected wins, or being unable to recognize bad decisions, as observed for variable lotteries with low potential gain, or actually experiencing a loss (Elliott et al., 2000; Critchley et al., 2002).

### Gain-related processing in the frontal cortex

Our data show that subjects were able to distinguish advantageous lotteries from disadvantageous ones based on our simple manipulation of expected gains and that choosing advantageously was associated with activity in ventromedial prefrontal cortex and the dorsal and ventral striatum. First, we observed increased activity in dorsal portions of the medial prefrontal cortex when subjects' choices resulted in wins. The region of the dorsal medial prefrontal cortex in which we report activity is thought to have similar value-related functions to those observed in ventral regions of the striatum. Studies have shown that the dorsal medial prefrontal cortex can track both receipt of current reward as well as expected reward (Knutson et al., 2001, 2003; Kuhnen and Knutson, 2005). Moreover, studies show that activity in regions of the dorsal medial prefrontal cortex is modulated by the level of expected value of the reward (Knutson et al., 2005; Luk and Wallis, 2009). Activity in the dorsal medial prefrontal cortex is also correlated with behavioral preferences, reflecting each individual's valuation of different options (McClure et al., 2004; Hare et al., 2009). Like the striatum, it should be noted that recent studies have also reported overlapping representations of both action and stimulus values in the dorsal medial prefrontal cortex (Chib et al., 2009; Glascher et al., 2009).

Although we observed increased activity in the OFC as a result of receiving a reward, we did not observe the activity we predicted would be associated with simple valuation in the comparison of all trials with high expected gains against trials with low expected gains. As discussed previously, activity in the OFC has been linked to the valuation of stimuli in decision making contexts in both humans and primates (Padoa-Schioppa and Assad, 2008; Padoa-Schioppa, 2009). Activity in neurons in the OFC also reflects subjects' willingness to pay to consume presented goods (Plassmann et al., 2007; FitzGerald et al., 2009) as well as self-reported experiences of pleasantness (Plassmann et al., 2008). Here, we found activation in the OFC when we separated trials not solely according to expected gains, but rather by a combination of potential gains and choice of the variable lottery (uncertain) vs. the constant lottery (certain). First, we compared trials in which the potential gain of the variable lottery was greater than that of the constant lottery and found increased activity for trials in which subjects chose to play the variable lottery over the safe lottery. Additionally, we found OFC activity associated with playing the variable lottery even when its potential gain was lower than the constant lottery.

One factor to consider is that this task was not new to the subjects at the time of the scanning session. It is possible that during pre-training subjects developed general representations of what an advantageous lottery was vs. a disadvantageous one based on value and preference, and only had to refine these representations in the scanner. Activity in the OFC may rely on a process of valuation that is more likely to occur when under states of uncertainty, as subjects must constantly try determine preference with only partial information regarding reward. This explanation would account for why we did not observe OFC activity associated with playing the safe lottery, as the value of this option was previously established.

### Role of the striatum

In addition to regions of the ventromedial frontal cortex, we found regions of the ventral and dorsal striatum that were modulated by expected gain and received reward. The ventral striatum was more active for wins than losses, which is consistent with this region playing a fundamental role in reward processing and reward prediction error (Schultz et al., 1997; Bayer and Glimcher, 2005). In the reinforcement learning framework, dopamine activity signals reward prediction error in which a reward that is better than expected will elicit a phasic burst of dopamine, a fully expected reward elicits no activity, and a reward that is worse than expected will produce a depression of dopaminergic firing (Schultz, 2002). Most notably, we observed greater gain-related activity in the ventral striatum when we compared trials in which the win was unexpected (the outcome of playing a variable lottery) or fully expected (the result of playing a certain lottery).

We found increased activation in regions of the putamen and posterior caudate nucleus for trials resulting in unexpected wins and activity in the posterior caudate for wins overall. Studies in non-human primates have suggested that neurons in these areas are involved in linking reward and motor behavior (Kawagoe et al., 1998; Lauwereyns et al., 2002; Ikeda and Hikosaka, 2003; Kobayashi et al., 2007). Moreover, activity in regions of the posterior caudate has been linked with successful learning of probabilistic reward-outcome associations (Seger and Cincotta, 2005, 2006; Foerde et al., 2006; Nomura et al., 2007). This pattern of basal ganglia recruitment is consistent with known patterns of corticostriatal interaction: the putamen is linked to primary motor and somatosensory cortex, and motor planning areas such as the premotor and supplementary motor cortices within the motor loop, which is thought to facilitate the selection and execution of appropriate motor responses during learning (Alexander et al., 1991; Lawrence et al., 1998; Haber et al., 2000, 2006; Seger, 2008). The posterior caudate nucleus interacts with the visual cortex in the occipital lobe, inferior temporal cortex, middle temporal cortex, and frontal eye fields of the frontal cortex to form the visual loop. Throughout the course of learning, it is believed that the visual loop helps relate various visual representations of stimuli to potential actions and rewards (Seger, 2008). Both the motor and visual corticostriatal loops are active during feedback-mediated learning tasks, such as categorization, in which the correct visual representation of the stimulus and the corresponding motor response must be selected to achieve a goal (Seger and Cincotta, 2005; Foerde et al., 2006; Cincotta and Seger, 2007). Therefore, it is believed that the visual and motor corticostriatal loops are involved in the formation of new stimulus-response associations, integrating new information with previously learned information and appropriate motor responses. Our results suggest that while subjects were making choices within various lotteries, they were also attempting to learn the most rewarding stimulus-response-outcome associations in a probabilistic learning environment.

## Conclusions

We identified different neural responses associated with linear and quadratic “inverted-U” functions sensitive to level of ambiguity. These results provide support for proposed theories of neural function under states of uncertainty that suggest ambiguity processing in the fronto–parietal–striatal network is greater at intermediate levels rather high levels. The graded coding of uncertainty we reported may reflect a unified treatment of risk and ambiguity as part of a general system evaluating uncertainty mediated by the DLPFC which recruits different regions of the prefrontal cortex as well as other valuation and learning systems according to the inherent difficulty of a decision. Finally, we showed that learning and valuation processes are modulated by expectancy and uncertainty; activity in regions related to the valuation of stimuli or options increased in situations where the decision making environment was uncertain

### Conflict of interest statement

The authors declare that the research was conducted in the absence of any commercial or financial relationships that could be construed as a potential conflict of interest.
